# Web-TCGA: an online platform for integrated analysis of molecular cancer data sets

**DOI:** 10.1186/s12859-016-0917-9

**Published:** 2016-02-06

**Authors:** Mario Deng, Johannes Brägelmann, Joachim L. Schultze, Sven Perner

**Affiliations:** Pathology of the University Hospital of Luebeck and Leibniz Research Center Borstel, Luebeck and Borstel, Germany; Leibniz Research Center Borstel, Borstel, Germany; Genomics and Immunoregulation, LIMES-Institute, University Bonn, Carl-Troll-Straße 31, 53115 Bonn, Germany; Department of Internal Medicine III Section of Hematology/Oncology, University Hospital of Bonn, Sigmund-Freud-Str. 25, 53127 Bonn, Germany

**Keywords:** TCGA, Cancer genomics, Statistics, Web application, Genomic data

## Abstract

**Background:**

The Cancer Genome Atlas (TCGA) is a pool of molecular data sets publicly accessible and freely available to cancer researchers anywhere around the world. However, wide spread use is limited since an advanced knowledge of statistics and statistical software is required.

**Results:**

In order to improve accessibility we created Web-TCGA, a web based, freely accessible online tool, which can also be run in a private instance, for integrated analysis of molecular cancer data sets provided by TCGA. In contrast to already available tools, Web-TCGA utilizes different methods for analysis and visualization of TCGA data, allowing users to generate global molecular profiles across different cancer entities simultaneously. In addition to global molecular profiles, Web-TCGA offers highly detailed gene and tumor entity centric analysis by providing interactive tables and views.

**Conclusions:**

As a supplement to other already available tools, such as cBioPortal (Sci Signal 6:pl1, 2013, Cancer Discov 2:401–4, 2012), Web-TCGA is offering an analysis service, which does not require any installation or configuration, for molecular data sets available at the TCGA. Individual processing requests (queries) are generated by the user for mutation, methylation, expression and copy number variation (CNV) analyses. The user can focus analyses on results from single genes and cancer entities or perform a global analysis (multiple cancer entities and genes simultaneously).

**Electronic supplementary material:**

The online version of this article (doi:10.1186/s12859-016-0917-9) contains supplementary material, which is available to authorized users.

## Background

With the ongoing decrease of cost for next generation sequencing and other high throughput molecular characterization methods, a vast amount of data sets are generated and provided for public access on web portals. In the field of cancer research, The Cancer Genome Atlas (TCGA) [1] data portal is the largest and most commonly used public resource, providing somatic mutation, gene expression, gene methylation and copy number variation (CNV) data sets, amongst others, for several thousands of tumor samples. Even though TCGA is a powerful and well-organized repository of molecular data types, the mining of its data is still limited. This is due to the fact that many tools (e.g. Excel or SPSS) commonly used by researchers outside the field of bioinformatics, are not capable of handling this vast amount of data. Moreover, the data sets provided often require preprocessing or further analysis, which may require advanced expertise in computational biology and computer programming. Other available tools, such as the cBioPortal, TCGA-Assembler [2] or Firebrowse (http://firebrowse.org/) show limitations when comparing multiple studies or require programming skills. Indeed cBioPortal can handle multiple studies at the same time, but is not able to incorporate them into a single analysis and does not provide any methylation analyses, while both modes are available with Web-TCGA. Further, TCGA-Assember does not include any graphical interface, which is an integral part of Web-TCGA. In addition, Firebrowse is only capable of displaying pre-calculated results, while Web-TCGA uses these data for further statistics and visualization., Therefore Web-TCGA is a fast and flexible web based software, which provides an infrastructure for comprehensive analyzing and visualizing the most common data types provided by TCGA. Web-TCGA is freely available and accessible to everyone and can also be run as a standalone application on every computer having the R programming language installed (see private instance below).

## Implementation

### Data integration

The data available at the TCGA is provided by the Firehose Pipeline of the Broad Institute [1] and was directly downloaded from this archive using the firehose_get script [3]. The data of the test instance is imported from the Firehose pipeline as of September 2014 and will be updated on a regular basis.

### Data processing

Within TCGA the data are provided on different levels, ranging from one to three, each indicating an increasing state of pre-processing and data aggregation. On level one only raw-data is provided, while level two is characterized by basic pre-processing or filtering, which depends on the data type (see below). Level three data are only available if segmented and interpreted results can be provided for that data type.

To keep the amount of data as small as possible and to reduce preprocessing and calculation time, Web-TCGA includes the highest data level available for each type. Web-TCGA directly makes use of somatic mutation data (level 2) and somatic CNV data (level 4, GISTIC2.0 output [4]), which do not require any further processing. For methylation and gene expression profiling, level 3 data is imported and processed as described below. For gene expression status two different preprocessing methods are available, namely RNA-SeqV1 (Reads Per Kilobase per Million, RPKM) and RNA-SeqV2 (RNA-Seq by Expectation Maximization, RSEM). We decided to use RNA-SeqV2, which takes transcript length into account and is suggested to provide more accurate results [5] for downstream analysis. Here, gene expression profiles are calculated using RSEM data. Due to the lack of normal samples, the relative expression for a specific gene is calculated by Web-TCGA using its expression status in a tumor sample of a given entity, compared to its average expression status in the remaining samples of the same entity [6]. For comparison one can choose whether to use the z-score or the fold change as basis for calculation. The z-score is defined as number of standard deviations above or below the mean of the gene's expression levels in the reference cohort.

For methylation data, we incorporated two modes of analysis into Web-TCGA. The first considers paired tumor/normal samples only, the second takes all samples of an entity into account. Within each mode a paired/unpaired Wilcoxon test is followed by a Benjamini-Hochberg p-value correction [7] to estimate differential methylation levels, as introduced by Hinoue et al. [8]. A p-value correction is necessary to correct for multiple hypothesis testing. Besides these test statistics, one can quantify the difference between tumor and control methylation values, which allows the user to estimate if a complete gene or parts of a gene is hyper- or hypomethylated. This second type of calculation is considered when rendering graphics, since p-values are not correlated with the direction of differential methylation.

### Private instance

For running a private instance, the source code can be downloaded from github (see paragraph Availability and Requirements) and the TCGA data can be downloaded using firehose_get by the user. Further it is possible to obtain the data using third party applications (such as TCGA-Assembler) as long as the TCGA default format is maintained. Afterwards the data is integrated using fully automated R scripts provided in the *pre-process_data* folder and becomes available after restarting the application.

## Results and Discussion

### The Web-TCGA user interface

Web-TCGA is semantically divided into a left and right hand work space. While the left one is used for user input only, the right one is used for navigation and output (see Additional file 1: Figure S1). In the right workspace, the red highlighted navigation bar is used to choose the type of data to analyze and supplies additional information about the methods used. Within each element of the navigation bar an additional navigation bar (highlighted in purple) is displayed for choosing the analysis method. This bar is specific to each data type. Below this second navigation bar, the yellow highlighted field is reserved for graphical output. In the left workspace, the user is supplied with the input menus (highlighted green), which are semantically divided by the type of input (highlighted dark blue).

### Visualization of global mutation profiling

**Fig. 1:**
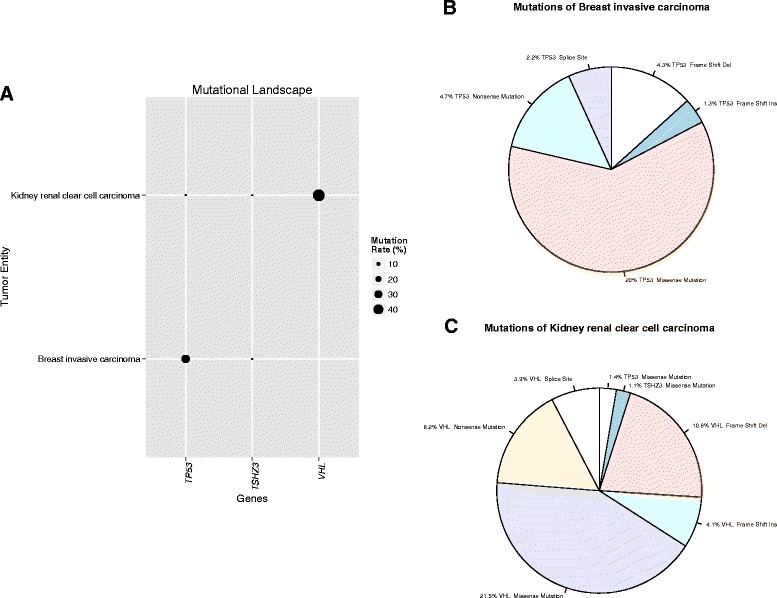
Illustration of mutational data. **a** Provides an overview of the global mutational profile of *TP53*, *TSHZ3* and *VHL* highlighting the cancer entity specific occurrence of mutations within BRCA and KIRC, while *TSHZ3*, as control, remains undetected. In **b** and **c**, the mutational profile is broken down into the type of predicted impact on the protein sequences. The slice size denotes the proportion of a mutation type in relation to all mutations. The given percentage denotes the proportion of a mutation type in relation to all tumor samples of a given entity

As proof of principle, we created global mutation profiles of several well-known cancer entity-specific alterations using Web-TGCA. For somatic mutation profiling, we tested *TP53* known to be associated with breast invasive carcinoma (TCGA abbreviation BRCA) but not kidney renal clear cell cancer (KIRC), *VHL* being associated with KIRC, but not BRCA, and *TSHZ3* shown to be absent in both entities. For BRCA, 992 samples from TCGA and for KIRC, 437 samples were included into our analysis. Figure 1a shows the visualization of the global mutation profile in Web-TCGA (including the cancer entities and genes selected by the user) of *TP53*, *VHL* and *TSHZ3* for these to tumor entities. As expected, the overall mutation rate for *TP53* is much higher in BRCA (33.1 %) than in KIRC (1.8 %), vice versa for *VHL* and extremely low for *TSHZ3* in both entities. To further illustrate the mutation frequency of these genes, we incorporated circle charts for a more detailed view (Fig. 1b and c). Here, the mutation frequencies of these three genes within each cancer entity are depicted, divided into the percentage of non-sense mutations, missense mutations, frame-shift deletions and insertions and splice sites alterations. Generally, all variant classes provided by the Firehose pipeline are considered, but not displayed if variants of this class are not present in the queried entities. Of great advantage, these graphs highlight that structure-changing variants of TP53 and VHL are highly associated with the BRCA and KIRC cancer types. These results are in high concordance with a recent study [9].

### Visualization of global methylation profiling

**Fig. 2:**
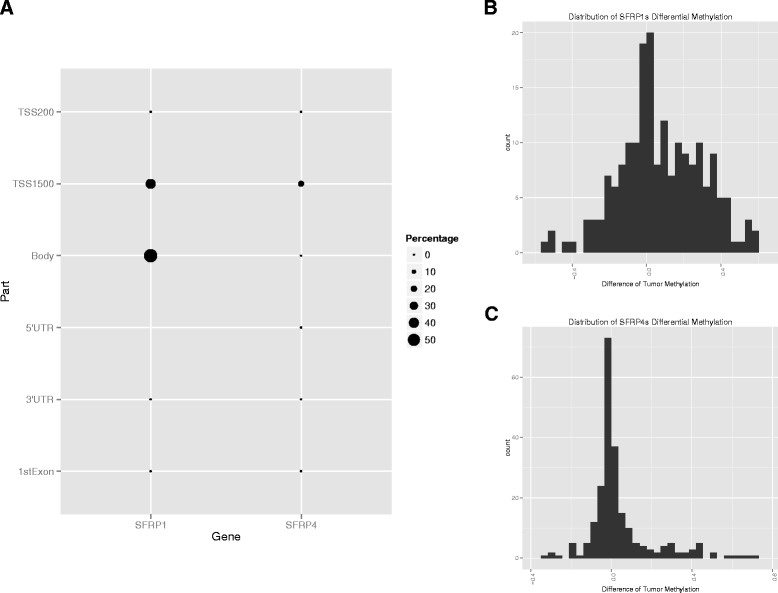
Illustration of methylation data. Different to all other analyses types for methylation data the global view is just provided for one cancer entity at a time, due to the fact that the gene is partitioned into different regions. **a** Provides a global profile of the methylation status in SFRP1 and SFRP4 in COAD, where SFRP1s is highly differential methylated in its body region and SFRP4, as control, remains mostly unmethylated. Further, **b** and **c** give an estimate of the differential methylation distribution, which also allows the detection of shifts. The x-axis shows difference of the ß-values of the methylation status between tumor and normal samples. The y-axis reflects the number of samples

To demonstrate the performance of Web-TCGA in methylation profiling, we analyzed the methylation status of *SFRP1* and *SFRP4* known to be highly differentially methylated in colon adenocarcinoma (COAD) [10]. In the gene bodies we could confirm that in almost all COAD samples *SFRP1* but not *SFRP4* was differentially methylated compared to normal controls (see Fig. 2a). For a more detailed illustration, Web-TCGA also provides differential methylation histograms (Fig. 2b and c). These histograms allow to estimate whether a gene is more hyper-, hypo-methylated or both (for the latter see Fig. 2c).

### Visualization of global expression profiling

**Fig. 3:**
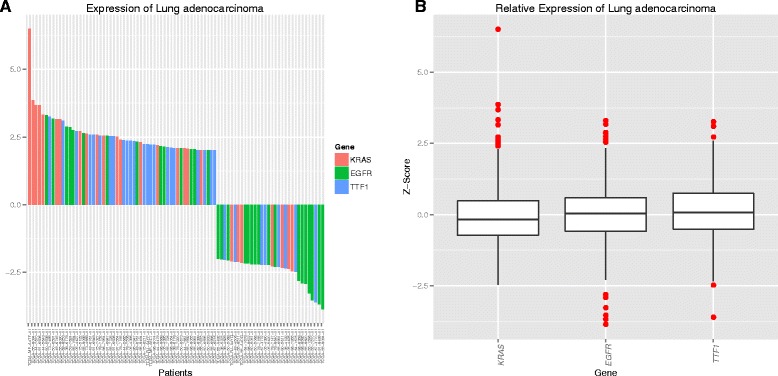
Illustration of expression data. To examine the global expression profile, the genes expression status in multiple cancer entities is rendered in the same way, as in Fig. 1a. Further, a waterfall plot, as shown in (**a**), is rendered for each cancer entity including the genes KRAS, EGFR and TTF1, outlining the patients exceeding a user given threshold for the genes expression on the x-axis, with the expression level on the y-axis. This plot is available in an entity (shown here) or a gene centric view. Further, Web-TCGA provides entity wise box plots (**b**), depicting an overview for the expression status of a cancer entity and enabling the identification of the expression patterns per cancer entity

For illustration of global expression data Web-TCGA provides an overview of the expression landscape of all available tumor entities and genes. To take the quantitative measure of expression data into account, Web-TCGA provides waterfall diagrams and box plots for illustration (Fig. 3a and b). In Fig. 3 we have chosen KRAS, EGFR and TTF1 known to be frequently expressed in lung adenocarcinoma (LUAD). As expected more samples show an over expression of these genes than an under expression.

### Visualization of CNVs

**Fig. 4:**
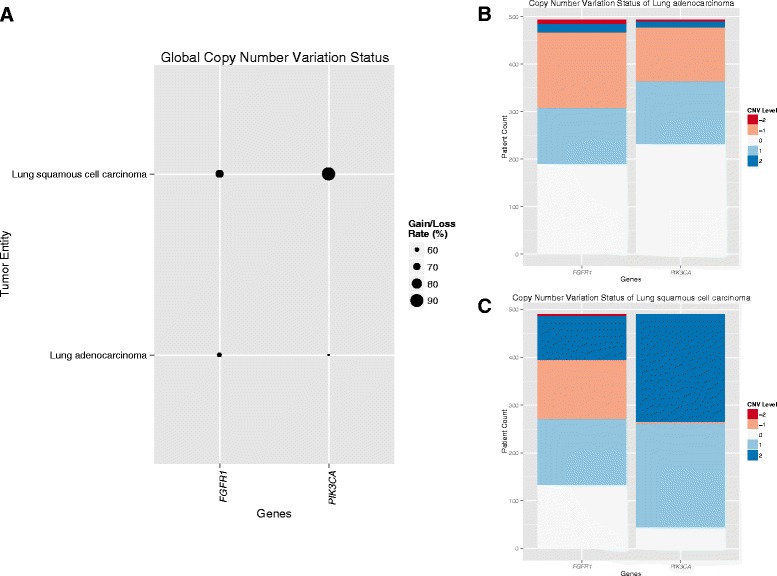
Illustration of CNV data. Just as for all other analyses types a global CNV profile is provided by Web-TCGA, as shown in **a** where the copy number status for the genes FGFR1 and PIK3CA within the entities LUSC and LUAD is shown, giving a first overview of the CNV profile, which is conducted of all samples harboring a gain or a loss. Furthermore, this profile can be created for all combinations of gains and losses alone, to distinguish the proportions of low and high level gains and losses. To further resolve these global profiles, Web-TCGA provides bar plots, as shown in **b** and **c**, for each entity chosen. These bar plots can be used to estimate the proportions of gains and losses within each entity in a much finer grain as in (**a**)

For handling CNV data GISTIC2.0 is used as basis. Each gene in each sample is characterized by the copy number variation value −2, −1, 0, 1 or 2, denoting homozygous deletion, heterozygous deletion, normal CN, low level and high level amplifications, respectively. In the global CNV view (Fig. 4a), Web-TCGA makes direct use of these categories and enables comparison of CNV status of all genes and entities. This view can also be restricted to one of the CNV values mentioned above. In the more detailed view, Web-TCGA allows an illustration of the absolute number of samples within each CNV value, for the genes and entities to be analyzed (Fig. 4b and c). Here, we compared the CNV categories of *FGFR1* and *PIK3CA* in Lung squamous cell carcinoma (LUSC) and Lung adenocarcinoma (LUAD). This comparison reflects the published results [11], where *PIK3CA* is highly amplified in more than 50 % of LUSC, but only in 4 % of LUAD. Furthermore, *FGFR1* is highly amplified in 72 % of all LUSC, but only in a small subset of LUAD, and of these with mainly low-level amplifications.

## Conclusion

Compared to existing tools such as cBioPortal [12, 13] and Firehose, Web-TCGA extends and complements available functionality with its ability to display global views that allow illustration and comparison of up to all genes across up to all tumor entities. Furthermore, Web-TCGA provides numerous more detailed views of molecular aberrations to facilitate in-depth analyses. Since these views can be generated in a gene or entity centric way the user can select to evaluate molecular aberrations of a given gene in up to all cancer entities and vice versa. Therefore Web-TCGA is a flexible and powerful open source software which can be accessed over the internet and can be installed on any computer. This software offers researchers a fast, simple and integrative access to large-scale studies of cancer genetics and provides features that are complementary and supplement to existing tools such as cBioPortal, Firebrowse and TCGA-Assembler. Similarly to cBioPortal, due to the usage of high-level data, all functionalities of Web-TCGA were implemented for real time usage. Therefor a response within seconds is guarantied. To gain a better understanding of Web-TCGAs functionalities and underline its capabilities we provide more examples for each analysis type in the supplemental material.

## Availability and requirements

- Project name: Web-TCGA
- Project homepage: https://github.com/mariodeng/web-TCGA
- Operating System: Platform independent
- Programming language: R 3.0.0
- Other requirements:
  - ○ firehose_get
  - ○ R packages: data.table, ggplot2 [14], markdown
- License: MIT

## Additional file

Additional file 1:
**The supplemental material includes additional examples and an introduction to the Web-TCGA user interface.** (DOCX 1522 kb)

## Abbreviations

BRCA: Breast invasive carcinoma
CNV: Copy number variation
COAD: Colon adenocarcinoma
KIRC: Kidney renal clear cell cancer
LUAD: Lung adenocarcinoma
LUSC: Lung squamous cell carcinoma
RPKM: Reads Per Kilobase per Million
RSEM: RNA-Seq by Expectation Maximization
TCGA: The Cancer Genome Atlas
