# Supplementary material for: Web-TCGA: an online platform for integrated analysis of molecular cancer data sets
Source: BMC Bioinformatics. 2016 Feb 6;17:72. doi: 10.1186/s12859-016-0917-9 (PMC4744375; doi:10.1186/s12859-016-0917-9)
Supplement: Additional file 1: — The supplemental material includes additional examples and an introduction to the Web-TCGA user interface. (DOCX 1522 kb) [file 12859_2016_917_MOESM1_ESM.docx]

1 The Web-TCGA user interface


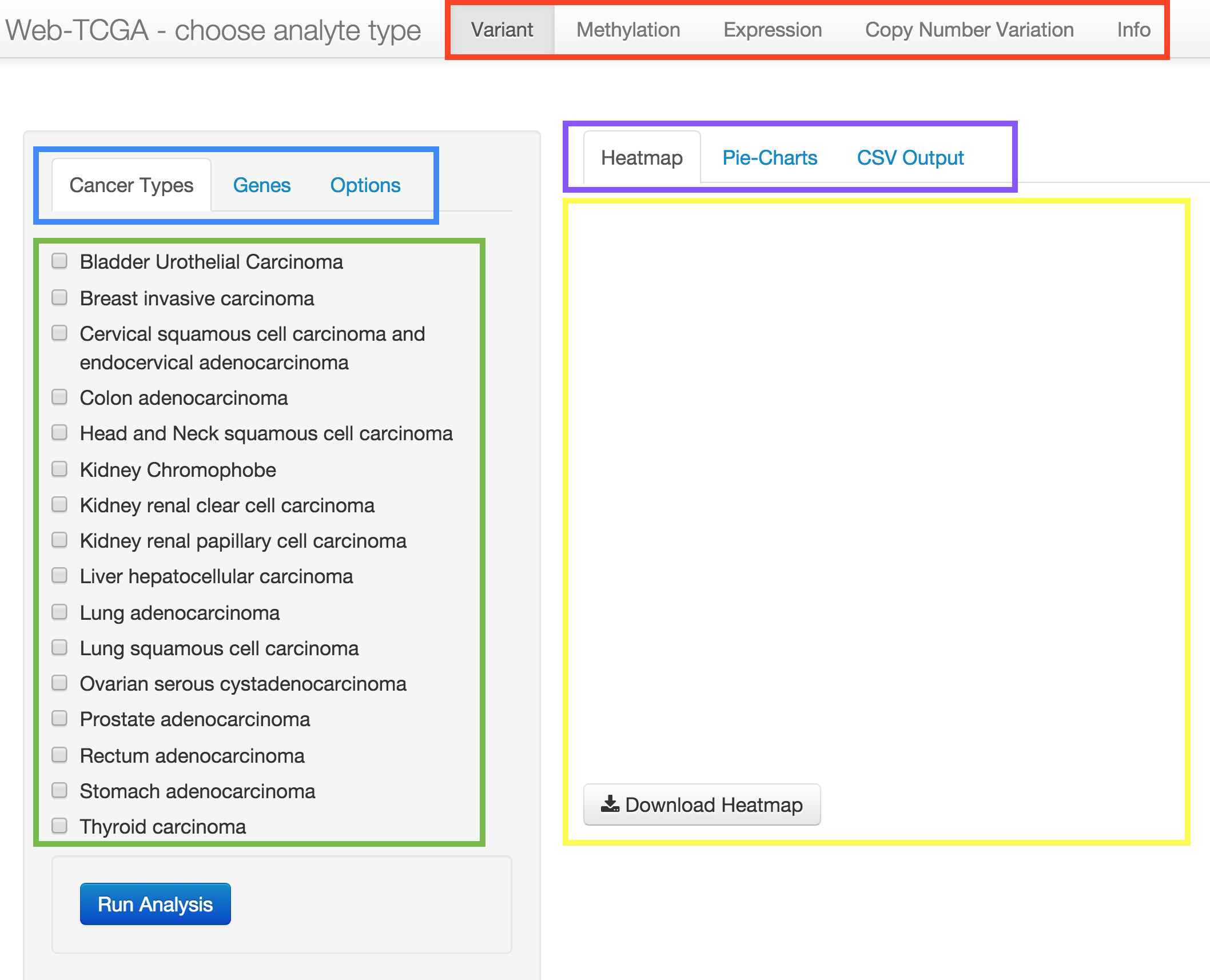


Web-TCGAs user interface is separated into three main parts. At the very top (red box) the user can choose which data type he wants to investigate. To the left hand side, the view is separated into a tabbed selection field (blue box), from where all analysis parameters (green box) are accessible. To the right hand side, within the yellow box, the results are displayed and a download button is provided.

2 Additional Examples

**2.1 Analyses of variants**

As an additional example we investigated the mutational profile of three genes, namely *APC*, *MLL3* and *NAV3*. Where *NAV3* and *MLL3* are known to be frequently mutated within lung adenocarcinomas, while it is also known that Bladder cancer is characterized by frequent *MLL3* mutations [1]. Here the gene *APC* and the breast cancer entity remain as a control. To further depict the global mutation profile shown in 2.1a, we plotted the proportional mutation rate in 2.1b, c and d, where the pie size indicated the proportion of mutation frequencies for the selected genes and their labeling the overall amount of mutations within the cohort.

**2.2 Methylation analyses**

To outline the methylation analysis in more detail, we have chosen the genes *RASSF1* and CDH1. Within the entity of prostate cancer it is assumed that *RASSF1* shows patterns of hyper- and hypomethylation, as suggested by Kim et al [2]. In 2.2a *RASSF1*s TSS200 and TSS1500 region appear with a significant number of more samples being differentially methylated, compared to CDH1. This is further illustrated in 2.2b where the distribution of *RASSF1*s methylation status is shifted to the right, where the distribution for *CDH1* in 2.2c remains centered.

**2.3 Gene Expression analyses**

Here we investigated the expression profile for *BRAF* and *AR* within prostate cancer and breast invasive carcinomas. While *BRAF* does barely occur to be differentially expressed in prostate cancer (2.3.f), a significant amount of samples being differentially expressed can be found within the cohort of breast cancer (2.3.e) and vice versa, which was suggested by Golub et al in 1999 [3]. For a more detailed view 2.3.c and 2.3.d show the expression of those samples being differentially expressed and exceeding a user defined threshold (here a Z-Score of 2), while, for additional analyses, the samples barcodes printed onto the X-axis. The same is done in 2.3.a and 2.3.b, where the plot is cancer entity orientated and samples, being differentially expressed, are color-coded gene wise.

**2.4 Copy Number Variation**

*ERBB2* and *WWOX* have previously been utilized for pan-cancer pattern discovery by Zack et al [4]. Here we could confirm their finding, suggesting that both genes suffer amplifications and loss within ovarian cancer and lung adenocarcinomas, but remains nearly unchanged in kidney renal clear cell carcinomas, as shown in 2.4.a. This finding can be deciphered into a per gene CNV profile, as displayed in 2.4.b, where both gene do not occur with any high- or low level amplification nor deletion. The copy number profile for the samples within ovarian cancer cohort is shown 2.4.c, where it clear that most of the samples suffer a low level deletion, for both genes. Within the lung adenocarcinoma cohort ~50% of all samples occur with a hyper- or hypomethylation. In contrast to ovarian cancer, here *ERBB2* occurs with mostly with low level amplifications, while there are barely any low level amplifications within the ovarian cancer cohort.

1. Kandoth C, McLellan MD, Vandin F, Ye K, Niu B, Lu C, Xie M, Zhang Q, McMichael JF, Wyczalkowski MA, others: **Mutational landscape and significance across 12 major cancer types**. *Nature* 2013, **502**:333–339.

2. Kim JH, Dhanasekaran SM, Prensner JR, Cao X, Robinson D, Kalyana-Sundaram S, Huang C, Shankar S, Jing X, Iyer M, Hu M, Sam L, Grasso C, Maher CA, Palanisamy N, Mehra R, Kominsky HD, Siddiqui J, Yu J, Qin ZS, Chinnaiyan AM: **Deep sequencing reveals distinct patterns of DNA methylation in prostate cancer.** *Genome Res* 2011, **21**:1028–1041.

3. Golub TR, Slonim DK, Tamayo P, Huard C, Gaasenbeek M, Mesirov JP, Coller H, Loh ML, Downing JR, Caligiuri MA, Bloomfield CD, Lander ES: **Molecular classification of cancer: class discovery and class prediction by gene expression monitoring.** *Science* 1999, **286**:531–537.

4. Zack TI, Schumacher SE, Carter SL, Cherniack AD, Saksena G, Tabak B, Lawrence MS, Zhsng C-Z, Wala J, Mermel CH, Sougnez C, Gabriel SB, Hernandez B, Shen H, Laird PW, Getz G, Meyerson M, Beroukhim R: **Pan-cancer patterns of somatic copy number alteration.** *Nat Genet* 2013, **45**:1134–1140.
